# Femoral nerve palsy following primary total hip arthroplasty with the direct anterior approach

**DOI:** 10.1371/journal.pone.0217068

**Published:** 2019-05-20

**Authors:** Chisato Hoshino, Daisuke Koga, Gaku Koyano, Yuki Yamauchi, Tomoko Sakai, Atsushi Okawa, Tetsuya Jinno

**Affiliations:** 1 Department of Rehabilitation Medicine, Tokyo Medical and Dental University, Bunkyo-ku, Tokyo, Japan; 2 Department of Orthopaedic Surgery, Tokyo Medical and Dental University, Bunkyo-ku, Tokyo, Japan; Consorci Parc de Salut MAR de Barcelona, SPAIN

## Abstract

Nerve palsy following total hip arthroplasty (THA) can have a serious effect on a patient`s functional prognosis and on cost-effectiveness, and it is the leading cause of THA-associated medical litigation. However, only a few studies focus on femoral nerve palsy (FNP) following THA with the direct anterior approach (DAA). Moreover, several studies have reported that THA with DAA may result in higher complication rates, particularly during the so-called ‘learning-curve period’ for the surgeon. This study aimed to identify the incidence of FNP following primary THA with DAA, to determine presumed etiologies through a retrospective investigation of FNP clinical courses following primary THA with DAA and to identify any relationship between the occurrence of FNP following primary THA with DAA and the surgeon’s experience of DAA. Since August 2007, DAA for primary THA was introduced in our institution. All 273 consecutive primary THAs with DAA (42 bilateral and 189 unilateral cases) between August 2007 and February 2014 were included in this study. All patients’ charts and radiographs were reviewed to identify cases with palsy and to retrieve related factors. In this study, FNP was defined as weakness of the quadriceps femoris (manual muscle test <3) with or without sensory disturbance over the anteromedial aspect of the thigh. The incidence of FNP following primary THA with DAA was 1.1% (3/273 joints). In all 3 cases, the motor deficit recovered completely within a year. Suspected causes of the palsy in the 3 cases were believed to be improper positioning of the anterior acetabular retractor, excessive leg lengthening, or unknown etiology. There was no significant relationship between palsy and surgeon’s experience of DAA. In THA with DAA for patients requiring major leg lengthening, the likelihood of FNP must be considered. To prevent FNP, the anterior acetabular retractor must be placed properly.

## Introduction

### Background

Total hip arthroplasty (THA) provides an excellent pain-relieving effect and improves the quality of life for patients with end-stage hip osteoarthritis. THA not only provides the abovementioned benefits, but also has been reported to be medically and economically cost-effective in recent years [1, 2]. However, THA may result in severe complications, such as nerve palsy, dislocation, infection, peri-prosthetic fracture, pulmonary thromboembolism, vascular disorder, and so on, that can seriously affect the patients’ functional prognosis. The occurrence of these complications may result in medical litigation. In fact, more than 75% to 90% of arthroplasty surgeons were reported to have been the subject of a malpractice lawsuit [3, 4, 5]. Nerve palsy is the most common reason for medical litigation after THA [3, 4, 5, 6, 7]. Bokshan et al. reported litigation associated with hip and knee arthroplasty. Overall, 15.0% of cases ended in settlement and 29.6% ended in physician loss. In the cases of nerve palsy, 10.3% of cases ended in settlement and 53.9% ended in physician loss, and the doctor`s win rate was low [3]. McWilliams et al. reported that the rate of physician`s payment was 46% in cases of nerve palsy after THA [7]. In these two reports, the average payment for nerve injury was USD 1,089,825 and UKP 116,800 [3, 7]. The incidence of peripheral nerve palsy following primary THA has been estimated to be from 0% to 4.0% [8, 9, 10]. Although it is a rare complication, nerve palsy is a serious possible potential complication following THA.

### Rationale

In response to the worldwide evolution of minimally invasive surgery (MIS), minimally invasive surgical approaches for THA have also been developed. The approach between the tensor fasciae latae and sartorius is conventionally known as the Smith-Petersen approach. From the early half of the 2000s, several studies reported modifications of this approach to MIS, such as the direct anterior approach (DAA), which has received much attention. With this approach, the sciatic and obturator nerves run not as close to the approach technique. However, the femoral and lateral femoral cutaneous nerves run closer to this approach. Therefore, the femoral and lateral femoral cutaneous nerves may be easily damaged during the surgery [11, 12]. Several studies examined lateral femoral cutaneous nerve palsy. The incidence varied from 0% to 81% among studies [13, 14, 15, 16, 17, 18]. Bhargava et al. and Goudling et al. reported that the presence of impaired sensation did not appear to affect the patients’ functional outcomes [13, 14]. There were several studies that reported femoral nerve palsy (FNP) as one of the complications following THA with DAA [16, 19, 20, 21, 22]. Although FNP affects the functional outcome more than the lateral femoral cutaneous nerve palsy, to our knowledge, only a few studies have focused on FNP following THA with DAA [16, 23]. Furthermore, several studies have reported that THA with DAA may result in higher complication rates, particularly during the so-called ‘learning-curve period’ [12, 24, 25].There is a paucity of studies which enable surgeons to know whether the incidence of FNP following THA with DAA in their institutions is large or small, or to know how to prevent FNP following THA with DAA. Strategies to reduce complications following THA may have equivalent or a greater effect on the cost and long-term effectiveness of THA than further enhancements in implant longevity [26].

### Purpose

The first purpose of this study was to identify the incidence of FNP following primary THA with DAA and to determine its presumed etiologies through a retrospective investigation of the clinical courses of FNP following primary THA with DAA. The second purpose of this study was to identify any relationship between the occurrence of FNP following primary THA with DAA and the surgeon’s experience of DAA. We hypothesized that the incidence of FNP following THA with DAA during the surgeon’s learning-curve period was higher than that after the learning-curve period.

## Materials and methods

This study was a case series. Approval was obtained from the institutional research ethics committee of Tokyo Medical and Dental University Hospital (No. M2000-1099, Postoperative clinical survey of total hip arthroplasty). All individuals included in this manuscript have provided written informed consent (as outlined in PLOS consent form) to publish the case details through preoperative informed consent process.

### Study subjects

We decided to initiate the use of DAA for THA from August 2007 onwards in response to the worldwide evolution of minimally invasive surgical approaches for THA. From August 2007 and February 2014, 1059 primary THAs (160 bilateral and 739 unilateral cases) were performed at our institution. A total of 273 joints (25.8%) were treated with DAA, 126 joints (11.9%) with the anterolateral approach (ALA), and 660 joints (62.3%) with the posterolateral approach (PLA). Although there were no definite inclusion or exclusion criteria for selecting DAA, we gradually extended the adaptation of DAA from simple cases (e.g. mild deformity) to complex cases (e.g. severe deformity, obesity, and severe contracture); the ratio of THA with DAA was 11.6% in 2007, but increased to 48.6% in 2014. All 273 consecutive primary THAs with DAA (42 bilateral and 189 unilateral cases) performed between August 2007 and February 2014 were included in this study.

### Description of treatment and surgery

In this study, nerve palsy was diagnosed through a neurological examination conducted by the surgeon. Nerve palsy was defined as weakness (manual muscle test [MMT] less than 3) of the innervated muscle with or without sensory disturbance (numbness or hypo/hyperaesthesia with clear laterality) of the peculiar rule domain. Preoperative check for palsy was routinely performed. Postoperative check for palsy was also routinely performed as soon as the spinal or general anesthesia wore off. In cases with epidural anesthesia, if palsy was suspected, reevaluation was performed several hours after the epidural anesthesia was discontinued. All surgeries were performed with uncemented technique. The patients were placed in the supine position on a standard operating table allowing hip extension. No intraoperative image intensifier was used [27]. In general, spinal anesthesia was used. However, in cases of intraoperative electrophysiological monitoring or difficulty with spinal anesthesia, general anesthesia was used. We used transcranial electric stimulation-induced motor-evoked potential (TCE-MEP) as the intraoperative electrophysiological monitoring method, when leg lengthening could reach more than 3 cm as determined by preoperative planning [28]. Case 3 was the only case whose preoperative planned leg lengthening was more than 3 cm in this study group. For postoperative pain control, subcutaneous patient-controlled analgesia (PCA) was used for unilateral patients, whereas epidural PCA was used for bilateral patients. Epidural anesthesia not only played a role in post-operative analgesia, but also enhanced the analgesic effect of the spinal anesthesia when the operative time became too long. PCA was discontinued on the first postoperative day. Surgeons included 2 experienced hip surgery consultants and 7 hip surgery trainees. Each surgeon began using DAA during this series. One of the 2 experienced hip surgery consultants (DK) participated in all of these cases. Pharmacological and mechanical prophylaxis against venous thromboembolism was routinely used postoperatively. Rehabilitation started on the second operative day with weight-bearing as tolerated.

### Data sources

All charts and radiographs of 273 joints were retrospectively reviewed to collect information about the presence of palsy, age, sex, body mass index [BMI], preoperative diagnosis, hip treatment history, preoperative range of flexion, the ratio of leg lengthening to spino-malleolar-distance (%SMD) and each surgeon’s experience. Hip treatment history included previous hip surgery and conservative treatment for pediatric hip disorders, such as orthosis or casting. Preoperative range of flexion was an index of flexibility. %SMD was defined as the ratio of leg lengthening calculated from the preoperative and postoperative radiographs to the preoperative affected side’s SMD, measured manually. We used %SMD to assess the risk of nerve palsy by leg lengthening, taking into consideration each patient’s height. Each surgeon’s experience was defined as the number of the individual surgeon’s experiences of THA with DAA at the time of the surgery. In this study, not only the incidence of FNP but also those of sciatic, obturator, and lateral femoral cutaneous nerve palsy were identified. However, the main focus of this study was FNP that caused apparent motor deficit (MMT less than 3) with or without sensory disturbance.

### Other methods

The incidence of FNP following primary THA with DAA was calculated. With regard to cases with FNP, their clinical courses were assessed, including the time when the palsy was discovered, when it recovered, additional examinations and therapies, and presumed etiologies of the palsy. The neurological function of the extremity was graded using a modified Sunderland scale [28, 29, 30]. The odds ratio of FNP following THA with DAA of the first 20 cases for a single surgeon to that of the subsequent cases was calculated. The first 20 cases were considered for the learning-curve period [24].

### Statistical analysis

Joints with missing data were excluded from the statistical analysis. StatView version 5.0 (SAS Institute Japan, Tokyo, Japan) was used for statistical processing. Fisher’s exact probability test was used for comparison testing. Where zero-cells caused problems with the computation of the odds ratio, 0.5 was added to all cells. The level of significance was set at 5%.

## Results

### The incidence of FNP following primary THA with DAA

No cases of palsy were seen preoperatively, including those patients with a hip treatment history.

Postoperative FNP following primary THA with DAA was found in 3 joints (3 cases). Thus, the incidence of FNP following primary THA with DAA was 1.1% (3/273 joints). There were no cases of sciatic nerve palsy, obturator nerve palsy, or FNP that caused only sensory disturbance following primary THA with DAA. The proportion of the cases with clinically apparent lateral femoral cutaneous nerve palsy following primary THA with DAA was 11.7% (32/273 joints). During the observed period, there was 1 joint of FNP following primary THA with ALA, and 1 joint of sciatic nerve palsy following primary THA with PLA.

### Presumed etiologies by retrospective investigation of the clinical courses of FNP following primary THA with DAA

All data needed for the statistical analysis could be obtained from the medical records of 264 of the 273 joints. Therefore, statistical analysis was performed for these 264 joints. There were no cases of palsy among the 9 joints that were excluded [S1 Dataset]. Characteristics of the study population are shown in Table 1.

**Table 1:** Characteristics of the study population.

Factors	All cases	Case 1	Case 2	Case 3
Age (years)	61.7±12.9^1^ (19–89)	57	51	50
Sex	Female 244 Male 20	Female	Female	Female
BMI	22.9±3.5 (15.3–35.4)	21.9	32.7	24.0
Diagnosis	Dysplastic OA^2^ 202 Osteonecrosis 36 Primary OA^2^ 7 RDC^3^ 7 RA^4^ 5 SIF^5^ 5 Post-traumatic OA^2^ 2	Dysplastic OA^2^	Dysplastic OA^2^	Dysplastic OA^2^
Hip treatment history^6^	Yes 34 No 230	Yes (casting)	Yes (casting)	Yes (orthosis)
Preoperative flexion (°)	90±18^1^ (20–120)	50	60	100
Leg lengthening^7^ (cm)	1.1±0.6^1^ (0–3.2)	1.6	3.0	2.5
%SMD^8^ (%)	1.4±0.8^1^ (0.0–4.4)	2.0	3.9	3.6
Each surgeon’s experience^9^	40.2±39.0^1^ (1–135)	4	7	17

^1^Values are presented as mean ± standard deviation (range).

^2^OA: osteoarthritis

^3^RDC: rapidly destructive coxarthropathy

^4^RA: rheumatoid arthritis

^5^SIF: subchondral insufficiency fracture

^6^Hip treatment history includes previous hip surgery and conservative treatment for pediatric hip disorders.

^7^% Leg lengthening was calculated from the preoperative and postoperative radiographs.

^8^%SMD is defined as the ratio of leg lengthening to spino-malleolar-distance

^9^Each surgeon’s experience is defined as the number of the individual surgeon’s experiences of THA with DAA at the time of the surgery

The majority of the patients were female and suffered from osteoarthritis due to various grades of dysplasia, which was typical in Japan [31]. Given that there were many patients with congenital dislocation of the hip, there were 34 joints with hip treatment history. Twenty two joints had a history of casting, whereas four joints had a history of orthosis. Two joints had a history of conservative therapy of which details were unclear. Four joints had a history of previous surgery (open reduction for traumatic dislocation, open reduction and internal fixation for femoral neck fracture, open reduction for congenital dislocation, and joint preservation surgery for dysplasia). Two joints had a history of conservative therapy for Perthes disease. All 3 cases of FNP had a hip treatment history. The average leg lengthening was 1.1±0.6 cm, and the leg lengthening of cases 2 and 3 were greater than the average plus two standard deviations.

#### Clinical course common in the three cases

FNP was diagnosed soon after the anesthesia wore off, and MMT of the quadriceps femoris at that time was 0. Postoperative transcranial magnetic stimulation-induced motor-evoked potential (TCM-MEP) was measured within several days after the operation. TCM-MEP of the quadriceps femoris of the affected side was obtained, although it was significantly lower than TCM-MEP of other muscles. Therefore, it was possible to judge that the femoral nerve was not completely lacerated. Postoperative CT and MRI revealed no lesion compressing the nerve, such as a hematoma, an osseous, or prosthetic prominence. Other etiologies, such as spinal disorders, were excluded clinically. In all cases, complete motor recovery was seen within a year (Table 2). The length of hospital stay of the 3 cases was longer than the average length in Japan, which is 21 days including the postoperative rehabilitation period (the length of stay is much longer in Japan than in Western countries, mainly due to differences in the health insurance system). Thirty days of hospital stay is not considered long in Japan when taking into consideration the complications.

**Table 2:** Clinical course of each case.

		Case 1	Case 2	Case 3
Length of postoperative hospital stay		30days	38days	26days
Walking aid	At discharge	T cane Knee flexion block brace	T cane Knee flexion block brace	T cane
	At 1 year after the operation	Free	T cane (due to knee arthritis)	Free
Modified Sunderland scale^1^	At 1 year after the operation	Grade 2	Grade 2	Grade 2
	At the latest observation (after the operation)	Grade 1 (10 years)	Grade 1 (10 years)	Grade 2 (6 years)

^1^Modified Sunderland scale: Grade 1, normal limb; Grade 2, mild motor weakness and/or dysesthesias; Grade 3, orthosis for ambulation, mild dysesthesias; Grade 4, walking restricted, moderate pain, limited occupation; Grade 5, grossly impaired motor function and/or severe pain.

#### Case 1

TCM-MEP of the quadriceps femoris gradually amplified with time. Etiology of the palsy could not be presumed.

#### Case 2

TCM-MEP of the quadriceps femoris gradually amplified with time. On the other hand, decrease of TCM-MEP of the quadriceps femoris with hip extension and knee flexion (femoral nerve stretch test) became apparent on the 8th postoperative day. Therefore, since excessive leg lengthening of 3.0 cm (3.9%SMD) might be a possible cause, revision surgery to shorten the leg was performed on the 10th postoperative day; leg shortening of 0.7 cm was achieved by replacing the cup and head. Although no electrical and clinical changes were seen immediately after the revision surgery, TCM-MEP of the quadriceps femoris recovered with time.

#### Case 3

**Fig 1:**
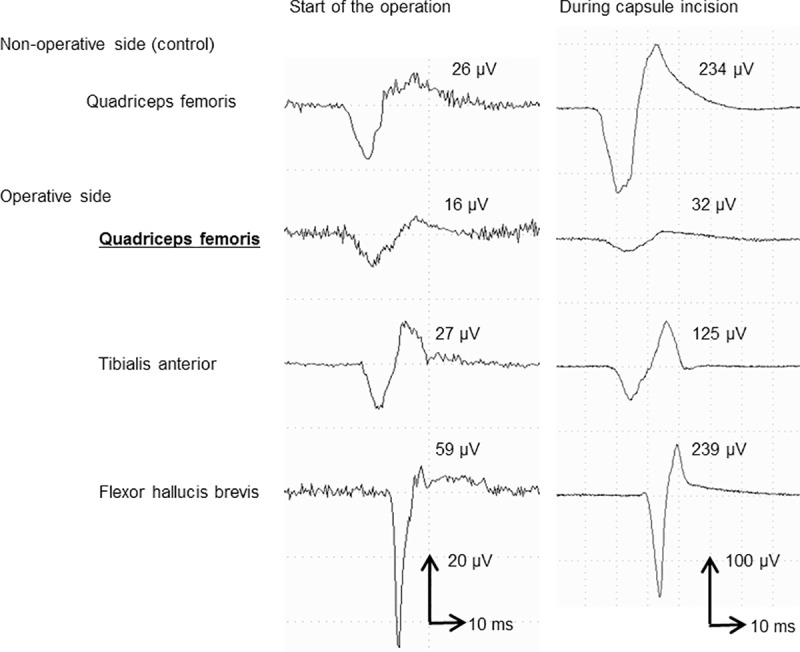
Intraoperative TCE-MEP in Case 3. TCE-MEP of the non-operative side’s quadriceps femoris (control) at this point increased to 900% compared with that at the start of the operation, and TCE-MEP of the operative side’s tibialis anterior and flexor hallucis brevis at this point increased to 463% and 405%, respectively, compared with that at the start of the operation. However, TCE-MEP of the operative side’s quadriceps femoris at this point increased only to 200% compared with that at the start of the operation.

Intraoperative transcranial electric stimulation-induced motor-evoked potential (TCE-MEP) was measured because preoperatively estimated leg lengthening was as long as 3.3 cm (4.8%SMD), which might have increased the risk of palsy. During anterior capsule incision with an electrosurgical knife, an unusual strong muscle contraction was observed. Accordingly, TCE-MEP was measured. TCE-MEP of the operative side’s quadriceps was lower than expected. Normally, TCE-MEP gets stronger because the muscle relaxant used at the induction of anesthesia is washed out with time. TCE-MEP of the non-operative side’s quadriceps femoris (control) at this point increased to 900% compared with that at the start of the operation, and TCE-MEP of the tibialis anterior and flexor hallucis brevis of the operative side at this point increased to 463% and 405%, respectively, compared with that at the start of the operation. However, TCE-MEP of the quadriceps of the operative side at this point increased only to 200% compared with that at the start of the operation (Fig 1). Positioning of the anterior acetabular retractor was examined, and it became apparent that the retractor was placed not directly on the anterior acetabular bony rim but on the iliacus muscle between the retractor and bony rim. Therefore, the retractor was removed. No recovery of the TCE-MEP was seen after several minutes of observation; however, the decreased amplitude of the quadriceps was reproducibly obtained, suggesting incomplete palsy. The retractor was replaced directly on the anterior acetabular bony rim. Afterwards, the unusual strong muscle contraction was not observed. Decreases in TCE-MEP with leg lengthening were not observed. Although we tried leg lengthening to the planned length, which was 3.3 cm (4.8%SMD), 2.5 cm (3.6%SMD) was the maximum lengthening achieved due to the tightness of the soft tissues. Given that recovery was clinically apparent soon after the operation, follow-up electrophysiological study was not performed.

### Other relevant findings

The odds ratio of FNP following THA with DAA of the first 20 cases for a single surgeon to that of the subsequent cases is shown in Table 3.

**Table 3:** The odds ratio of FNP following THA with DAA of the first 20 cases for a single surgeon to that of the subsequent cases.

		Palsy		Odds ratio = 8.1 95% C.I. 0.4–158.2 p = 0.10 N.S.
		+	-	
Each surgeon’s experience	First 20 cases	3	121	
	After 21 cases	0	140	

## Discussion

Although peripheral nerve palsy following THA is a rare complication, it is a serious one. The previous studies mostly focused on sciatic nerve palsy following the transtrochanteric or posterolateral approach [8, 9, 10, 32, 33]. Only a few studies have focused on FNP following THA with DAA [16, 23]

The reported incidence of FNP following THA is 0.01%-2.4% [8, 11, 19, 34]. Especially, the incidence of FNP following THA with DAA is 0% to 5% [16, 19]. The incidence in this study was 1.1%, which was about the same frequency as the incidence in previous studies with DAA.

In case 1, the exact cause was not apparent retrospectively. The exact etiologies of palsy cases are rarely identified with absolute certainty [9, 16, 32, 35]. In case 2, excessive leg lengthening might have been related to the FNP. As shown in previous studies examining other approaches [9, 32, 33, 36], major leg lengthening might be one of the risk factors for FNP following THA with DAA. The maximum amount that an extremity can be safely lengthened without a neurologic complication is not clear [32]. To prevent palsy with excessive leg lengthening, intraoperative electrophysiological monitoring is recommended when major leg lengthening is planned [11]. In case 2, actual leg lengthening (3.0cm) was longer than that planned preoperatively, which was 2.4 cm. In case 3, improper placement of the anterior acetabular retractor was thought to be the cause of the FNP. Previously, anatomical, radiological, and electrophysiological studies have reported that improper placement and/or excessive retraction of the anterior acetabular retractor are potential risk factors [8, 11, 23, 34, 37, 38, 39, 40, 41]. However, no study has reported that FNP was actually caused by improper placement of the anterior acetabular retractor. Case 3 was thought to be the first reported case of identified FNP that was actually an identified FNP caused by improper placement of the anterior acetabular retractor.

In all three cases in the present study, complete motor recovery was observed within a year, and recovery of electrophysiological functions was seen even within several weeks after the operation. In general, the recovery of FNP is more predictable than that of sciatic nerve palsy [9]. Siguier et al. reported that two cases of FNP following THA with DAA recovered completely within 9 months and 1 year [22]. One case that was reported by Hallert et al. also recovered completely at 6 months postoperatively [12]. These recovery patterns represent transient conduction block (neuropraxia).

Several studies have reported that THA with DAA may result in higher complication rates, particularly during the so-called ‘learning-curve period’ [12, 24, 25]. Although the FNP in this study occurred in the 4th and 7th cases for a hip surgery trainee and the 17th case for another hip surgery trainee, there was no significant relationship between the occurrence of palsy and the surgeon’ experience of DAA in this study (Table 3). However, no FNP following THA with DAA has been found in over 202 cases after this series until June 2018. The incidence of FNP decreased with time and, as of June 2018, it was 0.6 (3/475 joints). Johanson et al. reported that the incidence of nerve palsy could be reduced through increased experience of the surgeon [33]. At our institution, we experienced 3 cases of severe FNP at a relatively early stage after the introduction of DAA, which could be due to improper positioning of the anterior acetabular retractor and unintended leg lengthening. Therefore, we strengthened two preventive measures. One was to make sure to place the anterior acetabular retractor directly on the anterior acetabular bony rim, and the other was to ensure the actual leg lengthening equalled the planned leg lengthening. When the large leg lengthening was planned preoperatively, intraoperative electro physiological monitoring was performed to prevent palsy. It is thought that strengthened preventive measures might have led to the reduced incidence of FNP.

This study has several limitations. First, the incidence of FNP following THA with DAA might have been underestimated. In 1976, Weber et al. suggested that, when using electromyogram, the incidence of subclinical nerve abnormalities may be as high as 70% [35]. In 2018, Ishimatsu et al. also suggested that when using TCE-MEP, 77% of the patients showed a significant reduction of the femoral nerve amplitude with placement of the retractor on the anterior wall of the acetabulum [23]. There is no doubt that the clinically apparent palsy is the tip of the iceberg. Intra or postoperative electrophysiological testing was not performed for all cases in the present study. Therefore, subclinical nerve abnormalities may have been unrecognized. Moreover, there might have been a certain amount of unrecognized mild FNP after THA, because patients with mild palsy whose MMT of the quadriceps femoris is 4 are often able to stand and walk on flat surfaces using the usual postoperative assistive devices [11]. The three cases in this study had severe palsy as reflected by the MMT of the quadriceps femoris of 0. The incidence of FNP in this study was clinically apparent and actually affected patients’ functional outcomes. Although there were no definite criteria for selecting a surgical approach, there was a tendency to select DAA for simple cases, especially during the initiation of DAA. This is one of the possible factors that might have affected the incidence of FNP. Second, although we wanted to investigate the risk factors of FNP following THA with DAA through a statistical analysis, such as multivariate logistic regression, the number of cases with palsy was too small to be analysed statistically. In this study, we tried to determine the presumed etiologies through a retrospective investigation of the clinical courses and calculation of the odds ratio of FNP following THA with DAA of the first 20 cases for a single surgeon to that of the subsequent cases. Although it is outside the scope of this study, all 3 FNP cases in this study had a hip treatment history. There might be a relationship between occurrence of palsy and hip treatment history. Larger sample sizes are needed for more conclusive evidence.

## Conclusions

Postoperative FNP was identified in 3 joints (1.1%) out of the 273 consecutive cases of THA with DAA. In all 3 cases, complete motor recovery was seen within a year. Our results suggested that the likelihood for FNP must be considered in patients undergoing THA with DAA, especially those requiring major leg lengthening. Intraoperative proper placement of the anterior acetabular retractor was also thought to be critical in preventing FNP.

## Supporting information

S1 Dataset Minimal dataset.xlsx: The minimal data set, for the manuscript results presented here, is attached. (XLSX)
